# Abscisic Acid Induces Rapid Reductions in Mesophyll Conductance to Carbon Dioxide

**DOI:** 10.1371/journal.pone.0148554

**Published:** 2016-02-10

**Authors:** Giuseppe Sorrentino, Matthew Haworth, Said Wahbi, Tariq Mahmood, Shi Zuomin, Mauro Centritto

**Affiliations:** 1 Institute for Mediterranean Agriculture and Forest Systems, National Research Council, Via Patacca 85, 80056 Ercolano (NA), Italy; 2 Tree and Timber Institute, National Research Council (CNR - IVALSA), Via Madonna del Piano 10, 50019 Sesto Fiorentino (FI), Italy; 3 Laboratoire de Biotechnologie et Physiologie Végétale, Faculté des Sciences Semlalia, Université Cadi Ayyad, Boulevard My Abdellah BP 2390, Marrakech, Morocco; 4 Department of Environmental Sciences, Pir Mehr Ali Shah Arid Agriculture University, Murree Road, Rawalpindi, Pakistan; 5 Institute of Forest Ecology, Environment and Protection, Chinese Academy of Forestry, Key Lab on Forest Ecology and Environmental Sciences, State Forestry Administration, Beijing, 10091, China; Henan Agricultural Univerisity, CHINA

## Abstract

The rate of photosynthesis (*A*) of plants exposed to water deficit is a function of stomatal (*g*_s_) and mesophyll (*g*_m_) conductance determining the availability of CO_2_ at the site of carboxylation within the chloroplast. Mesophyll conductance often represents the greatest impediment to photosynthetic uptake of CO_2_, and a crucial determinant of the photosynthetic effects of drought. Abscisic acid (ABA) plays a fundamental role in signalling and co-ordination of plant responses to drought; however, the effect of ABA on *g*_m_ is not well-defined. Rose, cherry, olive and poplar were exposed to exogenous ABA and their leaf gas exchange parameters recorded over a four hour period. Application with ABA induced reductions in values of *A*, *g*_s_ and *g*_m_ in all four species. Reduced *g*_m_ occurred within one hour of ABA treatment in three of the four analysed species; indicating that the effect of ABA on *g*_m_ occurs on a shorter timescale than previously considered. These declines in *g*_m_ values associated with ABA were not the result of physical changes in leaf properties due to altered turgor affecting movement of CO_2_, or caused by a reduction in the sub-stomatal concentration of CO_2_ (*C*_i_). Increased [ABA] likely induces biochemical changes in the properties of the interface between the sub-stomatal air-space and mesophyll layer through the actions of cooporins to regulate the transport of CO_2_. The results of this study provide further evidence that *g*_m_ is highly responsive to fluctuations in the external environment, and stress signals such as ABA induce co-ordinated modifications of both *g*_s_ and *g*_m_ in the regulation of photosynthesis.

## Introduction

The rate of photosynthesis (*A*) in drought stressed plants is frequently constrained by the availability of carbon dioxide at the site of carboxylation. The concentration of CO_2_ within the chloroplast envelope (*C*_c_) is determined by stomatal (*g*_s_) and mesophyll (*g*_m_) conductance [1–3]. During water deficit, levels of ABA within the leaf are often enhanced by the transport of ABA from the roots to the shoots [4] and the conversion of ‘fixed’ glycosylated ABA stored within the vacuole to ‘free’ ABA in the cytosol [5, 6]. Stomatal conductance is negatively correlated to the concentration of ABA in the xylem [7] and leaf [8]. The effect of ABA on stomatal closure may be further enhanced as apoplastic ABA concentrates at the sites of evaporation close to the stomatal pores [9], and pH changes on a cellular level facilitate the movement of ABA to the guard cells [10]. Generally, stomatal and mesophyll conductance operate in tandem, showing marked reductions to drought [2, 11, 12]. It was shown that this tight coordination was controlled in an ultradian fashion in *Eucalyptus citriodora* plants subjected to different degrees of water deficit [13]. However, despite transport of CO_2_ across the mesophyll layer representing the largest resistance step in the uptake of CO_2_ for photosynthesis and a critical component in the drought stress response of plants, the response of *g*_m_ to ABA is not clear.

Mesophyll conductance is determined by physical [14–16] and biochemical [17, 18] factors. Any reduction in leaf turgor could alter physical resistances, such as apoplastic space and the porosity of the cell wall encountered by CO_2_ during transport from the sub-stomatal air-space to the chloroplast [9, 19, 20]. It is therefore necessary to distinguish between the physical influence of changes in leaf turgor experienced during drought, and any direct biochemical effects of ABA on *g*_m_. Relatively few studies have investigated the effect of ABA on *g*_m_, and have produced contrasting results; possibly due to the experimental approaches utilised to modify [ABA], either through increased endogenous production of ABA or exogenous application e. g. [21, 22], and the methods employed to measure *g*_m_ e.g. [23, 24, 25]. Exogenous application of ABA to hydroponically grown soybean (*Glycine max* L.) and tobacco (*Nicotiana tabaccum* L.) resulted in reduced *g*_s_ and *g*_m_ values over the course of ten days [3]. Identical reductions of both *g*_s_ and *g*_m_ were observed in cut-leaves of drought stressed and well-watered wild-type and ABA deficient mutants of tobacco (*Nicotiana plumbaginifolia*) two hours after exposure to a solution containing ABA [9]. The addition of ABA to the nutrient solution of ‘sand-grown’ sunflowers (*Helianthus annuus* L.) also reduced canopy-level *g*_m_ after three days [26]. In contrast, perlite grown sunflower plants supplied with an exogenous ABA solution showed reduced *g*_s_, but no alteration of *g*_m_ after three days [25]. Furthermore, no difference was observed in *g*_m_ values of wild-type and ABA insensitive mutants of *Arabidopsis thaliana* [27].

The disparity in responses of *g*_m_ to ABA may be related to interspecific differences in drought stress physiology. For example, concomitant reductions in both *g*_s_ and *g*_m_ are frequently observed in response to water-deficit e.g. [2]. However, in drought tolerant plants, persistent long-term water deficit may result in an enhancement of *g*_m_ in comparison to the early stages of drought, whilst *g*_s_ remains low [28, 29]. One other potential explanation is the methodology employed to gauge *g*_m_. It is not possible to measure *g*_m_ directly, and all approaches involve a number of assumptions and potential uncertainties [23]. Mesophyll conductance can be calculated through simultaneous measurement of leaf gas exchange and chlorophyll fluorescence parameters (the variable J method) [30, 31], determination of leaf gas exchange and carbon isotope discrimination [32, 33] or analysis of the curve of the photosynthetic response to increased [CO_2_] in the internal sub-stomatal air-space (*C*_i_) [34]. It has been suggested that stomatal closure during drought stress may alter rates of photosynthetic ‘recapture’ of CO_2_ released during photorespiration, affecting the measurement of levels of respiration in the light (*R*_d_) and thus the determination of *g*_m_ using the variable J method [23, 35]. However, observations of *g*_m_ sensitivity and insensitivity to ABA have been recorded using both the variable J method [3, 9, 25] and the carbon isotopic discrimination approach [9, 25, 26]. This may suggest that it is not possible to fully account for the disparity in *g*_m_ responses to ABA due to uncertainties with the methodological approach utilised to characterise *g*_m_. Furthermore, analysis of the effects of drought on *g*_m_ levels in rice (*Oryza sativa*) using both the variable J method and analysis of carbon isotope discrimination in recently synthesised sugars method indicated that the variable J technique was equally effective as the carbon isotopic discrimination approach in gauging *g*_m_ at low levels of water availability, but crucially produced more robust results under well-watered control conditions [2].

Abscisic acid plays a fundamental role in the drought stress response of plants. Given the wide-range of *g*_m_ responses to ABA reported in the literature over different timescales, we investigated *g*_m_ sensitivity to exogenous ABA application in four commercially important woody species. To characterise the direct biochemical response of *g*_m_ to ABA, measurements were taken over a short time period (four hours) to minimise any physical effects that may be associated with leaf water status that could affect the movement of CO_2_ from the sub-stomatal air-space to the chloroplast envelope. This study aimed to: i) investigate the effect of exogenous ABA on stomatal and mesophyll conductance over a four hour time-course; ii) characterise any interspecific variations in the *g*_m_ response of the four species to ABA; iii) assess diffusive constraints imposed by *g*_s_ and *g*_m_ following ABA treatment that determine rates of photosynthesis.

## Materials and Methods

### Plant material, growing conditions and ABA treatment

Cherry (*Prunus avium* L.), black poplar (*Populus nigra* L.), olive (*Olea europaea* L.) and rose (*Rosoideae rosa*, hybrid tea rose “Camp David“) were grown in a greenhouse at the National Research Council, Monterotondo, Rome, Italy. The plants were approximately one year-old and grown in comparatively large 6 dm^3^ pots, where they did not experience root-restriction, containing a sand-perlite mixture (1:3) under natural sunlight and photoperiod from June to August. The respective daily maximum and minimum air temperatures were 38 and 20°C. To avoid any water and nutrient limitation, the saplings were watered every other day to pot water capacity and fertilised once a week, with Hoagland nutrient solution to supply nutrients at free access rates [36].

The evening prior to measurement the plants were watered to pot water capacity. The next morning branches were cut under distilled water; control treatment branches remained in distilled water, and *cis-trans* ABA (99% purity, Sigma) was added to the water of branches subject to ABA treatment. An ABA solution of 10^−4^ M concentration was used. Simultaneous measurement of gas exchange and chlorophyll fluorescence was then performed every hour over a four hour period between 08:00 and 12:00 in a well ventilated air-conditioned room at 25°C with control and ABA-treated shoots placed under a metal halide light emitting 800 μmol m^-2^ s^-1^ PPFD.

### Gas exchange and fluorescence measurements

Leaf gas exchange and fluorescence parameters of the central leaf section were simultaneously measured using a LI-6400-40 leaf chamber fluorometer (Li-Cor, Inc., Nebraska, USA) equipped with a 2 cm^2^ cuvette. One branch for the ABA and control treatments was taken from each of four plants, with the youngest fully expanded leaf from four branches measured for each treatment. The measurements were made at a saturating photon flux density (PPFD) of 1600 μmol m^-2^s^-1^ measured using the internal quantum sensor within the leaf chamber and leaf temperature of 25°C for all four species. Leaves were exposed to a contaminant and pollutant free flux of synthetic air, composed of a mixture of nitrogen (80%), O_2_ (20%) and CO_2_ (385 ppm). The relative humidity of the air flow (500 μmol s-1) was maintained at 40–50%. To reduce diffusion leaks through the chamber gasket [37], a supplementary external chamber gasket composed of the same polymer foam was added to create an interspace between the two gaskets (i.e. a double-gasket design with a 5 mm space separating the internal and external gaskets). Then the CO_2_ and H_2_O gradients between the in-chamber air and pre-chamber air were minimized by feeding the IRGA exhaust air into the interspace between the chamber and the pre-chamber gaskets [38].

The variable J method has proven to be effective in determining *g*_m_ in both well-watered and drought stressed rice varieties [2], we therefore chose this approach to assess the effect of ABA on *g*_m_ in the four plant species. Mesophyll conductance was calculated using the variable J method involving simultaneous measurements of gas-exchange and chlorophyll fluorescence parameters as described by Harley et al. (30) and Loreto et al. (31) (Eqs 1 and 2):

$$g_{m}=\frac{A}{C_{i}-\frac{\Gamma *\left[J_{\text{F}}+8*\left(A+R_{d}\right)\right]}{J_{\text{F}}-4*\left(A+R_{d}\right)}}$$ (1)

where the electron transport rate (*J*_F_) is calculated from fluorescence [39]:

$$J_{F}=PPFD*\frac{\Delta F}{F_{m}}*\alpha *\beta$$ (2)

where *F*_m_ is the fluorescence maximum and the partitioning factor (*β*) between photosystems I and II was considered to be 0.5 and leaf absorbance (*α*) (0.85) [40].

The CO_2_ compensation point to photorespiration (Γ*) was measured on individual attached leaves of intact plants by increasing *C*_i_ at four different levels of photosynthetically active radiation [41, 42]. Levels of respiration in the light (*R*_d_) were analysed using the Kok method [43, 44]; and respiration in the dark (*R*_n_) was measured by switching off the light in the cuvette, when CO_2_ release from the leaf had become stable for approximately five to ten minutes this was recorded and considered to represent *R*_n_ [45]. Values of Γ*, *R*_d_ and *R*_n_ used in the calculation of *g*_m_ utilising the variable J method are given in Table 1. Total conductance to CO_2_ (*g*_tot_) was calculated as:

$$g_{\text{tot}}=\left[g_{\text{s}}*g_{\text{m}}\right]/\left[g_{\text{s}}+g_{\text{m}}\right]$$ (3)

**Table 1:** Values of the CO_2_ compensation point to photorespiration (Γ*), respiration in the light (*R*_d_) and respiration in the dark (*R*_n_) used to calculate *g*_m_ levels of the four plant species using the variable J method [30, 31]. ± indicates one standard deviation.

Species	*R*_n_ (μmol m^-2^s^-1^)	*R*_d_ (μmol m^-2^s^-1^)	Γ* (μmol mol-1)
Cherry	1.97 ± 0.28	1.40 ± 0.13	46.6 ± 2.77
Olive	1.72 ± 0.10	1.28 ± 0.09	58.4 ± 2.59
Poplar	1.80 ± 0.09	1.06 ± 0.12	45.5 ± 3.72
Rose	1.94 ± 0.20	1.24 ± 0.10	55.3 ± 3.59

### Leaf water status

Immediately following the gas exchange measurements, each leaf was detached and weighed to determine leaf fresh mass (*F*_M_). The leaves were then placed in a plastic bag and with the cut-end submerged in distilled water and allowed to rehydrate in darkness at 5°C for 18 hours. After rehydration the leaves were dried using paper towels to remove any water on their surfaces, and then the leaves were weighed to determine the saturated mass (*S*_M_). Leaves were then dried at 80°C for 48 hours to measure dry mass (*D*_M_). The relative water content (RWC) of each leaf was then calculated as follows [46]:

$$\text{RWC}=\left[F_{\text{M}}-D_{\text{M}}\right]/\left[S_{\text{M}}-D_{\text{M}}\right]$$ (4)

### Statistical analyses

Statistical analyses were performed using SPSS 20 (IBM, New York, USA). A one-way ANOVA with LSD *post-hoc* test was used to assess differences in variance between samples subjected to control conditions and ABA treatment. A significant difference between treatments was assumed to occur at a *P*-value <0.05. Linear regression was used to investigate potential relationships between conductance to CO_2_ and *A* in the four plant species under control conditions and ABA treatment.

## Results

**Fig 1:**
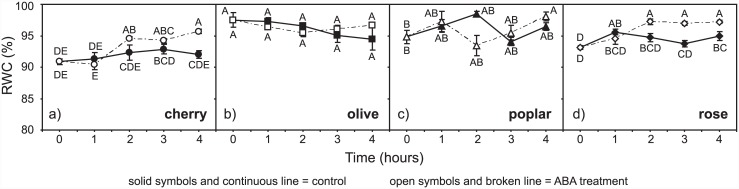
Relative water content values of control and ABA treated leaves of cherry (a), olive (b), poplar (c) and rose (d) over the four hour measurement period. Solid symbols and continuous line indicate control samples; open symbols and broken line indicate ABA treatment. Error bars indicate one standard error either side of mean. Different letters indicate significant differences between datasets based upon one-way ANOVA and LSD *post-hoc* test.

Following abscission of the cuttings, the RWC of the leaves was recorded at each hourly measurement interval to assess any alteration in leaf water content that may have influenced *g*_m_ via a change in turgor. The RWC of control cherry leaves remained within the range of 88.7–94.1% throughout the measurement period (Fig 1a). However, after two hours the mean RWC of leaves exposed to exogenous ABA increased from 90.9% to 95.7%; a significant difference between the RWC values of the control and ABA treated leaves only became apparent after four hours of exposure (one-way ANOVA LSD *post-hoc* test, F_1,6_ = 25.954; *P* = 0.00223). The RWC of rose leaves exhibited a similar pattern, with the mean RWC of ABA treated leaves exhibiting an increase from 94.6% to 97.2% after two hours. However, RWC parameters of control leaves also showed a slight increase during the measurement period over a range of 92.2 to 96.7%. Statistically significant differences were observed in the RWC values of control and ABA treated rose leaves after two hours (one-way ANOVA LSD *post-hoc* test, F_1,6_ = 9.416; *P* = 0.0220) and this persisted for the remainder of the measurement period (Fig 1d). The RWC of poplar leaves showed no significant decrease between the control and ABA treatments, although RWC rose slightly by 1.6–3.4% over the four hour measurement period (Fig 1c). The RWC values of olive leaves displayed no significant change during the four hour measurement period, and no significant treatment effect associated with the application of ABA (Fig 1b).

**Fig 2:**
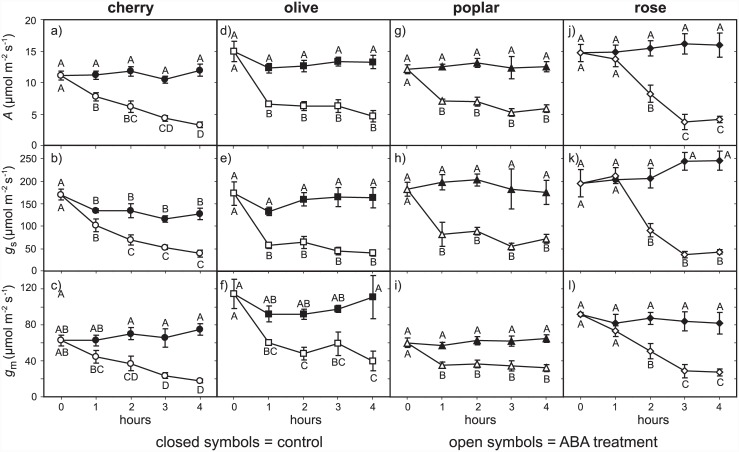
Time course *A*, *g*_s_ and *g*_m_ responses of cherry (a-c), olive (d-f), poplar (g-i) and rose (j-l) cuttings to application of exogenous ABA solution. Solid symbols indicate control samples; open symbols indicate ABA treatment. Error bars indicate one standard error either side of mean. Different letters indicate significant differences between datasets based upon one-way ANOVA and LSD *post-hoc* test.

**Fig 3:**
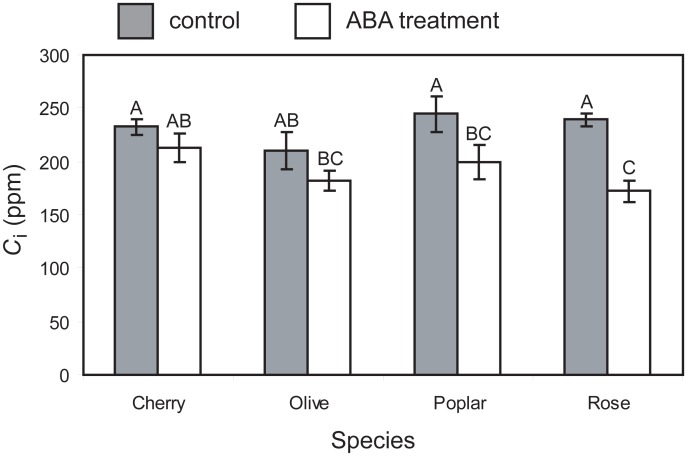
Internal sub-stomatal concentrations of CO_2_ (*C*_i_) of control and ABA treated cuttings after four hours. Grey indicates control; white indicates ABA treatment. Error bars indicate one standard error either side of mean. * indicates significant difference between control and ABA treatment values using a one-way ANOVA (cherry, F_1,6_ = 1.501, *P* = 0.266; olive, F_1,6_ = 2.020, *P* = 0.205; poplar, F_1,6_ = 8.998, *P* = 0.0240; rose, F_1,6_ = 34.991, *P* = 0.00104). Different letters indicate significant differences between datasets based upon one-way ANOVA and LSD *post-hoc* test.

Exposure to exogenous ABA induced consistent declines in *A* in all four of the species studied. The control plants showed no declines in *A* that may have been associated with branch abscission (Fig 2). These reductions in *A* following exposure to ABA corresponded to identical declines in *g*_s_ and *g*_m_. However, the rate of reduction in *g*_s_ and *g*_m_ values differed between the four species. Cherry exhibited a consistent decline in *A*, *g*_s_ and *g*_m_ throughout the measurement period following ABA treatment (Fig 2a–2c). Olive and poplar displayed rapid reductions in *g*_s_ (olive: -66.7%; poplar– 54.8%) and *g*_m_ (olive: -47.3%; poplar– 40.9%) in the first hour after exposure to exogenous ABA, before maintaining relatively stable *g*_s_ and *g*_m_ values for the remainder of the measurement period. In contrast, rose does not show a decline in levels of *A*, *g*_s_ and *g*_m_ in the first hour after ABA treatment, before exhibiting a rapid decline in the second hour (*A*: -44.2%; *g*_s_: -53.5%; *g*_m_: -44.5%) then stabilising in the third and fourth hour (Fig 2j–2l). The lower levels of conductance to CO_2_ and *A* following four hours of ABA treatment led to decreases in *C*_i_ of 8.4 to 28.0% in all four plant species. However, this reduced *C*_i_ was statistically significant only in poplar and rose (Fig 3).

**Fig 4:**
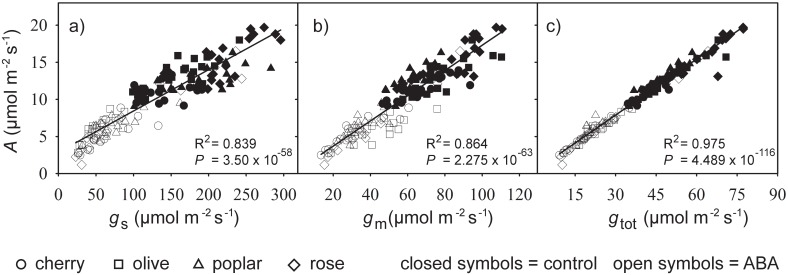
Relationship of *A* to *g*_s_ (a), *g*_m_ (b), and *g*_tot_ (c) in control (closed symbols) and ABA treated (open symbols) cuttings of cherry (circle symbol), olive (square symbol), poplar (triangle symbol) and rose (diamond symbol) after four hours of treatment. Line indicates best fit determined by linear regression: stomatal conductance (F_1,142_ = 740.620; R^2^ = 0.839; *P* = 3.350 x 10–58), mesophyll conductance (F_1,142_ = 901.489; R^2^ = 0.864; *P* = 2.275 x 10^−63^), and; total conductance to CO_2_ (F_1,142_ = 5618.153; R^2^ = 0.975; *P* = 4.488 x 10−^116^).

Photosynthetic rates of the four species were largely determined by conductance to CO_2_ (Fig 4). Under control conditions, rose generally exhibited the highest levels of *A*, *g*_s_ and *g*_m_, while cherry displayed the lowest. However, after four hours of ABA treatment, rose and cherry showed identical photosynthetic rates and levels of conductance to CO_2_. The leaves treated with exogenous ABA exhibited lower levels of *g*_s_, *g*_m_ and *g*_tot_ that correspond to lower *A*. The relationship between *A* and conductance to CO_2_ was most robust when *g*_s_ and *g*_m_ were combined to determine *g*_tot_ (Fig 4c).

## Discussion

Abscisic acid plays a fundamental role in plant responses to water-deficit. The effect of ABA on *g*_m_ is poorly characterised in comparison to the influence of ABA on stomatal behaviour e.g. [4, 47, 48]. This study demonstrated that exogenous ABA produced a rapid reduction in *g*_m_ values within two hours of application in all of the four species studied. These declines in *g*_m_ corresponded to reductions in *g*_s_, analogous to co-ordination of *g*_s_ and *g*_m_ observed during drought [2, 12, 49] and as circadian pattern [13]. The reductions in *g*_s_ following ABA application are indicative of stomatal sensitivity to ABA and active physiological stomatal behaviour in all four of the plant species studied [50, 51]. Photosynthesis was closely related to conductance to CO_2_ in the control and ABA treated cuttings (Fig 4), suggesting that ABA determined *A* through its action upon *g*_s_ and *g*_m_. The speed of response of *g*_m_, *g*_s_ and *A* to ABA application varied between the four species, possibly due to differences in water transport e.g. [52] affecting the uptake and movement of ABA, or biochemical differences in the effect of ABA signalling between the plants e.g. [53, 54].

Mesophyll conductance is composed of physical and chemical components that determine the movement of CO_2_ from the internal sub-stomatal air-space to inside the chloroplast envelope [55]. Increased RWC in ABA treated cherry and rose (Fig 1), possibly due to stomatal closure reducing transpirative water-loss from the leaf, may have altered the physical properties of the leaf and thus affected *g*_m_ indirectly. However, in the evening prior to abscission the plants were watered to pot capacity, and all of the leaves exhibited high RWC values that correspond to full leaf turgor [56], likely suggesting that the proportionally small percentage increases of 2–3% in RWC following ABA application did not induce significant alteration of leaf structural properties. It is noteworthy that significant differences in the RWC of control and ABA treated leaves of cherry only became apparent after two hours, whereas *g*_m_ values in ABA treated leaves declined significantly within one hour (Fig 2c); suggesting that the initial declines in following ABA application were solely the result of biochemical changes, and not due to alteration of the physical properties of leaves associated with a change in foliar water status.

The effect of ABA on *g*_m_ observed in this study was rapid, occurring within one hour of exposure in three of the four species studied. Short-term fluctuations in *g*_m_ have also been recorded in response to [CO_2_] [37], salinity [1, 57], light quality [58, 59], light intensity [37] and temperature [18], and have been ascribed to the action of carbonic anhydrase and cooporins that transport CO_2_ across the plasma membrane [17, 60–65]. The conversion of gaseous CO_2_ to aqueous carbonic acid (H_2_CO_3_) represents one of the largest resistance steps encountered in photosynthetic CO_2_-uptake [66]. The results of this study may suggest that ABA acts to reduce the activity of cooporins involved in the transport of CO_2_. Cooporins belong to a group of proteins known as aquaporins, that also facilitate the movement of water across membranes [67]. As foliar [ABA] increased, the activity of aquaporins have been shown to decline during drought stress [68, 69], and induce stomatal closure through increased guard cell permeability and reduced water movement across the membrane [70]. Exogenous application of ABA to fully hydrated leaves achieved similar results [71]. However, increased [ABA] may also enhance the expression of certain aquaporins to enhance drought tolerance through increased water transport [72].

It is unclear whether alterations in *g*_m_ are a by-product of the *g*_s_ response, or whether the two processes are linked by co-ordinated physiological signalling [73]. Instantaneous increases in *C*_i_ applied to plants grown under optimal conditions induce reductions in *g*_m_, as the limiting effect of CO_2_ availability on *A* declines at higher *C*_i_ [37]. This would suggest that if the *g*_m_ responses observed following ABA treatment in the present study were solely the result of reduced *C*_i_ due to stomatal closure, an increase in *g*_m_ might be expected. However, reduced *g*_m_ was recorded in all four species after application of exogenous ABA; furthermore, only two of the species exhibited significant declines in *C*_i_ after ABA treatment (Fig 3). Application of the same concentration of ABA used in this study to the roots of sunflower did not affect *g*_m_ but did reduce *g*_s_. The relationship between *g*_m_ and *C*_i_ in ABA treated sunflower was also identical to control plants; showing a positive correlation between *g*_m_ and *C*_i_ at concentrations above 200 ppm [25]. However, detached leaves of wild-type tobacco plants when treated with exogenous ABA exhibited a positive *g*_m_—*C*_i_ relationship at sub-ambient *C*_i_, whilst the control counterparts showed a negative *g*_m_—*C*_i_ relationship [73]. Furthermore, *g*_m_ response to *C*_i_ has been shown to occur on a shorter timescale than *g*_s_ response to *C*_i_ in tobacco, wheat and in both wild-type and mutant *Arabidopsis thaliana* that lacked the capacity for stomatal closure [74]. This may suggest that the concomitant declines in *g*_s_ and *g*_m_ observed in this study following ABA treatment are the result of a shared signalling mechanism regulating rates of stomatal and mesophyll conductance to CO_2_ [73] in addition to any subsequent effects on *g*_m_ associated with stomatal closure [73–75], respiration [23] or photorespiration [24].

It is noteworthy that the effect of ABA on *g*_m_ observed in the present study occurs more rapidly than previously reported e.g. [3, 9, 25, 26]. Increased foliar [ABA] following drought stress induces stomatal closure to restrict water-loss from the internal leaf [76]. It would initially appear incongruous to reduce transport of CO_2_ across the mesophyll layer in concert with decreased *g*_s_ to minimise water-loss from the internal leaf [73]; as a high *g*_m_ to *g*_s_ ratio would permit the maintenance of a degree of CO_2_ uptake and enhanced water use efficiency during drought [27]. However, the rapid declines in *g*_m_ after application with exogenous ABA may indicate a selective advantage of reduced conductance to CO_2_ across the mesophyll during episodes of water-deficit. A decline in the activity of cooporins responsible for the transport of CO_2_ may reduce energy consumption [77]. Higher cellular [78] and apoplastic [79] ABA may also be associated with changes in pH that alter membrane properties through the action of proteins such as aquaporins [80], thus affecting *g*_m_ [17]. Nonetheless, it is presently unclear as to the nature of the mechanisms responsible for such rapid alterations in *g*_m_ following ABA treatment or their functional significance. Further analysis of the expression of cooporin protein RNA may elucidate the biochemistry underlying this response e.g. [81]. Application with exogenous ABA induced declines in *g*_m_ within one hour in three of the species studied (and within two hours in the remaining species, rose); this would suggest that ABA has a clear effect on *g*_m_, and the interaction of ABA and *g*_m_ plays an important role in plant drought stress response.

## Conclusion

The results of this study show consistent reductions in *A*, *g*_s_ and *g*_m_ values of all four plants following exposure to exogenous ABA. Photosynthesis in the plant species was positively related to the availability of CO_2_ within the chloroplast envelope (Fig 4). The observed declines in *g*_m_ occurred on a shorter timescale than those reported in previous studies; suggesting that ABA serves to induce rapid reductions in *g*_m_ following exposure to water deficit. These falls in *g*_m_ values occurred prior to any significant alteration in RWC; suggesting that they are not associated with physical effects of increased turgor affecting the permeability of cell membranes, and are instead the result of alteration to the biochemical properties of the interface between the mesophyll and the internal sub-stomatal air-space. The reductions in *g*_m_ recorded in the present study are unlikely to be the by-product of stomatal closure causing a decrease in *C*_i_, as plants exposed to ABA exhibited reduced *g*_m_ rather than increased conductance to counter lower availability of CO_2_ in the sub-stomatal air-space. The effect of ABA on *g*_m_ is likely through the diminished activity of cooporins and carbonic anhydrase, whether these responses act on a dose-dependent basis or occur over different timescales is currently unclear. However, the findings of the present study indicate that ABA functions by inducing rapid reductions in *g*_m_ that are associated with concomitant declines in *g*_s_ as part of a co-ordinated gas exchange response to water deficit.

## References

[1] Centritto M, Loreto F, Chartzoulakis K. The use of low [CO_2_] to estimate diffusional and non-diffusional limitations of photosynthetic capacity of salt-stressed olive saplings. Plant, Cell and Environment. 2003;26(4):585–94. 10.1046/j.1365-3040.2003.00993.x

[2] Lauteri M, Haworth M, Serraj R, Monteverdi MC, Centritto M. Photosynthetic diffusional constraints affect yield in drought stressed rice cultivars during flowering. PloS one. 2014;9(10):e109054. 25275452 10.1371/journal.pone.0109054 PMC4183539

[3] Flexas J, Ribas-Carbó M, Bota J, Galmés J, Henkle M, Martínez-Cañellas S, et al Decreased Rubisco activity during water stress is not induced by decreased relative water content but related to conditions of low stomatal conductance and chloroplast CO_2_ concentration. New Phytol. 2006;172(1):73–82. 10.1111/j.1469-8137.2006.01794.x 16945090

[4] Davies WJ, Zhang JH. Root signals and the regulation of growth and development of plants in drying soil. Annu Rev Plant Physiol Plant Mol Biol. 1991;42:55–76. PubMed PMID: ISI:A1991FP08300004.

[5] Lee KH, Piao HL, Kim H-Y, Choi SM, Jiang F, Hartung W, et al Activation of glucosidase via stress-induced polymerization rapidly increases active pools of abscisic acid. Cell. 2006;126(6):1109–20. 16990135 10.1016/j.cell.2006.07.034

[6] Xu Z-J, Nakajima M, Suzuki Y, Yamaguchi I. Cloning and characterization of the abscisic acid-specific glucosyltransferase gene from adzuki bean seedlings. Plant Physiol. 2002;129(3):1285–95. 12114582 10.1104/pp.001784 PMC166522

[7] Zhang J, Davies W. Changes in the concentration of ABA in xylem sap as a function of changing soil water status can account for changes in leaf conductance and growth. Plant, Cell and Environment. 1990;13(3):277–85.

[8] Thomas DS, Eamus D. The influence of predawn leaf water potential on stomatal responses to atmospheric water content at constant *C*_i_ and on stem hydraulic conductance and foliar ABA concentrations. J Exp Bot. 1999;50(331):243–51. 10.1093/jxb/50.331.243

[9] Mizokami Y, Noguchi K, Kojima M, Sakakibara H, Terashima I. Mesophyll conductance decreases in the wild type but not in an ABA-deficient mutant (aba1) of *Nicotiana plumbaginifolia* under drought conditions. Plant, Cell Environ. 2015;38(3):388–98. 10.1111/pce.12394 PubMed PMID: MEDLINE:24995523. 24995523

[10] Cowan IR, Raven JA, Hartung W, Farquhar GD. A possible role for abscisic acid in coupling stomatal conductance and photosynthetic carbon metabolism in leaves. Funct Plant Biol. 1982;9(4):489–98. 10.1071/PP9820489

[11] Flexas J, Ribas-Carbó M, Diaz-Espejo A, Galmēs J, Medrano H. Mesophyll conductance to CO_2_: current knowledge and future prospects. Plant, Cell Environ. 2008;31(5):602–21. 10.1111/j.1365-3040.2007.01757.x 17996013

[12] Centritto M, Lauteri M, Monteverdi MC, Serraj R. Leaf gas exchange, carbon isotope discrimination, and grain yield in contrasting rice genotypes subjected to water deficits during the reproductive stage. J Exp Bot. 2009;60(8):2325–39. 10.1093/jxb/erp123 PubMed PMID: WOS:000266348800010. 19443613

[13] Brilli F, Tsonev T, Mahmood T, Velikova V, Loreto F, Centritto M. Ultradian variation of isoprene emission, photosynthesis, mesophyll conductance, and optimum temperature sensitivity for isoprene emission in water-stressed *Eucalyptus citriodora* saplings. J Exp Bot. 2013;64(2):519–28. 10.1093/jxb/ers353 PubMed PMID: WOS:000313618900012. 23293347

[14] Adachi S, Nakae T, Uchida M, Soda K, Takai T, Oi T, et al The mesophyll anatomy enhancing CO_2_ diffusion is a key trait for improving rice photosynthesis. J Exp Bot. 2013;64(4):1061–72. 10.1093/jxb/ers382 PubMed PMID: WOS:000316003600021. 23349143

[15] Evans J, Caemmerer S, Setchell B, Hudson G. The relationship between CO_2_ transfer conductance and leaf anatomy in transgenic tobacco with a reduced content of rubisco. Funct Plant Biol. 1994;21(4):475–95. 10.1071/PP9940475

[16] Tomás M, Flexas J, Copolovici L, Galmés J, Hallik L, Medrano H, et al Importance of leaf anatomy in determining mesophyll diffusion conductance to CO_2_ across species: quantitative limitations and scaling up by models. J Exp Bot. 2013;64(8):2269–81. 10.1093/jxb/ert086 23564954 PMC3654418

[17] Hanba YT, Shibasaka M, Hayashi Y, Hayakawa T, Kasamo K, Terashima I, et al Overexpression of the barley aquaporin HvPIP2;1 increases internal CO_2_ conductance and CO_2_ assimilation in the leaves of transgenic rice plants. Plant and Cell Physiology. 2004;45(5):521–9. 10.1093/pcp/pch070 15169933

[18] Bernacchi CJ, Portis AR, Nakano H, von Caemmerer S, Long SP. Temperature response of mesophyll conductance. Implications for the determination of Rubisco enzyme kinetics and for limitations to photosynthesis in vivo. Plant Physiol. 2002;130(4):1992–8. 10.1104/pp.008250 PubMed PMID: WOS:000179990100044. 12481082 PMC166710

[19] Grassi G, Magnani F. Stomatal, mesophyll conductance and biochemical limitations to photosynthesis as affected by drought and leaf ontogeny in ash and oak trees. Plant, Cell and Environment. 2005;28(7):834–49.

[20] Parida AK, Das AB, Mittra B. Effects of salt on growth, ion accumulation, photosynthesis and leaf anatomy of the mangrove, *Bruguiera parviflora*. Trees. 2004;18(2):167–74. 10.1007/s00468-003-0293-8

[21] Tardieu F, Lafarge T, Simonneau T. Stomatal control by fed or endogenous xylem ABA in sunflower: interpretation of correlations between leaf water potential and stomatal conductance in anisohydric species. Plant, Cell and Environment. 1996;19(1):75–84.

[22] Correia MJ, Pereira JS, Chaves MM, Rodrigues ML, Pacheco CA. ABA xylem concentrations determine maximum daily leaf conductance of field-grown *Vitis vinifera* L. plants. Plant, Cell and Environment. 1995;18(5):511–21. 10.1111/j.1365-3040.1995.tb00551.x

[23] Pons TL, Flexas J, von Caemmerer S, Evans JR, Genty B, Ribas-Carbo M, et al Estimating mesophyll conductance to CO_2_: methodology, potential errors, and recommendations. J Exp Bot. 2009;60(8):2217–34. 10.1093/jxb/erp081 19357431

[24] Gilbert ME, Pou A, Zwieniecki MA, Holbrook NM. On measuring the response of mesophyll conductance to carbon dioxide with the variable J method. J Exp Bot. 2012;63(1):413–25. 10.1093/jxb/err288 21914657 PMC3245476

[25] Vrabl D, Vaskova M, Hronkova M, Flexas J, Santrucek J. Mesophyll conductance to CO_2_ transport estimated by two independent methods: effect of variable CO_2_ concentration and abscisic acid. J Exp Bot. 2009;60(8):2315–23. 10.1093/jxb/erp115 PubMed PMID: WOS:000266348800009. 19433478

[26] Schäeufele R, Santrucek J, Schnyder H. Dynamic changes of canopy-scale mesophyll conductance to CO_2_ diffusion of sunflower as affected by CO_2_ concentration and abscisic acid. Plant, cell and environment. 2011;34(1):127–36. 10.1111/j.1365-3040.2010.02230.x 21029117

[27] Flexas J, Niinemets Ü, Gallé A, Barbour M, Centritto M, Diaz-Espejo A, et al Diffusional conductances to CO2 as a target for increasing photosynthesis and photosynthetic water-use efficiency. Photosynthesis Res. 2013;117(1–3):45–59. 10.1007/s11120-013-9844-z 23670217

[28] Aganchich B, Wahbi S, Loreto F, Centritto M. Partial root zone drying: regulation of photosynthetic limitations and antioxidant enzymatic activities in young olive (*Olea europaea*) saplings. Tree Physiology. 2009;29(5):685–96. 10.1093/treephys/tpp012 PubMed PMID: WOS:000265850500006. 19324696

[29] Shi Z, Cheng R, Liu S, Sorrentino G, Centritto M. Carbon assimilation, δ13C and water relations of *Elaeagnus angustifolia* grown at two groundwater depths in the Minqin desert, China. Plant Biosystems. 2008;142(3):525–32.

[30] Harley PC, Loreto F, Dimarco G, Sharkey TD. Theoretical considerations when estimating the mesophyll conductance to CO_2_ flux by analysis of the response of photosynthesis to CO_2_. Plant Physiol. 1992;98(4):1429–36. 10.1104/pp.98.4.1429 PubMed PMID: WOS:A1992HR53200034. 16668811 PMC1080368

[31] Loreto F, Harley PC, Dimarco G, Sharkey TD. Estimation of mesophyll conductance to CO_2_ flux by three different methods. Plant Physiol. 1992;98(4):1437–43. 10.1104/pp.98.4.1437 PubMed PMID: WOS:A1992HR53200035. 16668812 PMC1080369

[32] Evans JR, Sharkey TD, Berry JA, Farquhar GD. Carbon isotope discrimination measured concurrently with gas exchange to investigate CO_2_ diffusion in leaves of higher plants. Aust J Plant Physiol. 1986;13(2):281–92 PubMed PMID: WOS:A1986D134400008.

[33] Scartazza A, Lauteri M, Guido MC, Brugnoli E. Carbon isotope discrimination in leaf and stem sugars, water-use efficiency and mesophyll conductance during different developmental stages in rice subjected to drought. Aust J Plant Physiol. 1998;25(4):489–98. PubMed PMID: WOS:000074645400011.

[34] Ethier GJ, Livingston NJ. On the need to incorporate sensitivity to CO_2_ transfer conductance into the Farquhar—von Caemmerer—Berry leaf photosynthesis model. Plant, Cell and Environment. 2004;27(2):137–53. 10.1111/j.1365-3040.2004.01140.x

[35] Tholen D, Ethier G, Genty B, Pepin S, Zhu X-G. Variable mesophyll conductance revisited: theoretical background and experimental implications. Plant, Cell and Environment. 2012;35(12):2087–103. 10.1111/j.1365-3040.2012.02538.x 22590996

[36] Hoagland DR, Arnon DI. The water-culture method for growing plants without soil. Circular California Agricultural Experiment Station. 1950;347(2nd edit).

[37] Flexas J, Diaz-Espejo A, Galmés J, Kaldenhoff R, Medrano H, Ribas-Carbo M. Rapid variations of mesophyll conductance in response to changes in CO_2_ concentration around leaves. Plant, Cell and Environment. 2007;30(10):1284–98. 10.1111/j.1365-3040.2007.01700.x 17727418

[38] Rodeghiero M, Niinemets Ü, Cescatti A. Major diffusion leaks of clamp-on leaf cuvettes still unaccounted: how erroneous are the estimates of Farquhar et al. model parameters? Plant, Cell Environ. 2007;30(8):1006–22. 17617828 10.1111/j.1365-3040.2007.001689.x

[39] Genty B, Briantais J-M, Baker NR. The relationship between the quantum yield of photosynthetic electron transport and quenching of chlorophyll fluorescence. Biochimica et Biophysica Acta (BBA)—General Subjects. 1989;990(1):87–92. 10.1016/S0304-4165(89)80016-9

[40] Laisk A, Loreto F. Determining photosynthetic parameters from leaf CO_2_ exchange and chlorophyll fluorescence (ribulose-1, 5-bisphosphate carboxylase/oxygenase specificity factor, dark respiration in the light, excitation distribution between photosystems, alternative electron transport rate, and mesophyll diffusion resistance. Plant Physiol. 1996;110(3):903–12. 12226229 10.1104/pp.110.3.903 PMC157790

[41] Laisk A. Kinetics of photosynthesis and photorespiration in C3 plants. Nauka Moscow (in Russian). 1977.

[42] Brooks A, Farquhar GD. Effect of temperature on the CO_2_/O_2_ specificity of ribulose-1,5-bisphosphate carboxylase/oxygenase and the rate of respiration in the light. Planta. 1985;165(3):397–406. 10.1007/BF00392238 24241146

[43] Kok B. A critical consideration of the quantum yield of *Chlorella* photosynthesis. Enzymologia. 1948;13:1–56.

[44] Yin X, Sun Z, Struik PC, Gu J. Evaluating a new method to estimate the rate of leaf respiration in the light by analysis of combined gas exchange and chlorophyll fluorescence measurements. J Exp Bot. 2011;62:3489–99. 10.1093/jxb/err038 21382918 PMC3130174

[45] Haworth M, Moser G, Raschi A, Kammann C, Grünhage L, Müller C. Carbon dioxide fertilisation and supressed respiration induce enhanced spring biomass production in a mixed species temperate meadow exposed to moderate carbon dioxide enrichment. Funct Plant Biol. 2016;43:26–39. 10.1071/FP15232 32480439

[46] Diaz-Pérez JC, Shackel KA, Sutter EG. Relative water content and water potential of tissue 1. J Exp Bot. 1995;46(1):111–8. 10.1093/jxb/46.1.111

[47] Snaith P, Mansfield T. Stomatal sensitivity to abscisic acid: can it be defined? Plant, Cell and Environment. 1982;5(4):309–11.

[48] Tardieu F, Davies WJ. Stomatal response to abscisic acid is a function of current plant water status. Plant Physiol. 1992;98(2):540–5. 16668674 10.1104/pp.98.2.540 PMC1080223

[49] Sun P, Wahbi S, Tsonev T, Haworth M, Liu S, Centritto M. On the use of leaf spectral indices to assess water status and photosynthetic limitations in *Olea europaea* L. during water-stress and recovery. Plos One. 2014;9(8):e105165 10.1371/journal.pone.0105165 25136798 PMC4138116

[50] Haworth M, Killi D, Materassi A, Raschi A. Co-ordination of stomatal physiological behavior and morphology with carbon dioxide determines stomatal control. Am J Bot. 2015;102(5):677–88. 10.3732/ajb.1400508 26022482

[51] Chater C, Gray JE, Beerling DJ. Early evolutionary acquisition of stomatal control and development gene signalling networks. Curr Opin Plant Biol. 2013;16(5):638–46. 10.1016/j.pbi.2013.06.013 23871687

[52] Gleason SM, Butler DW, Ziemińska K, Waryszak P, Westoby M. Stem xylem conductivity is key to plant water balance across Australian angiosperm species. Funct Ecol. 2012;26(2):343–52. 10.1111/j.1365-2435.2012.01962.x

[53] McAdam SAM, Brodribb TJ. Fern and lycophyte guard cells do not respond to endogenous abscisic acid. The Plant Cell Online. 2012;24(4):1510–21. 10.1105/tpc.112.096404 PMC3398560 22517320

[54] Blackman P, Davies W. The effects of cytokinins and ABA on stomatal behaviour of maize and *Commelina*. J Exp Bot. 1983;34(12):1619–26.

[55] Warren CR, editor Stand aside stomata, another actor deserves centre stage: the forgotten role of the internal conductance to CO_2_ transfer 14th International Congress of Photosynthesis; 2007 Jul 22–27; Glasgow, Scotland: Oxford University Press. 10.1093/jxb/erm245 17975206

[56] Bramley H, Ehrenberger W, Zimmermann U, Palta J, Rüger S, Siddique KM. Non-invasive pressure probes magnetically clamped to leaves to monitor the water status of wheat. Plant Soil. 2013;369(1–2):257–68. 10.1007/s11104-012-1568-x

[57] Loreto F, Centritto M, Chartzoulakis K. Photosynthetic limitations in olive cultivars with different sensitivity to salt stress. Plant Cell and Environment. 2003;26(4):595–601. 10.1046/j.1365-3040.2003.00994.x PubMed PMID: WOS:000182009600011.

[58] Loreto F, Tsonev T, Centritto M. The impact of blue light on leaf mesophyll conductance. J Exp Bot. 2009;60(8):2283–90. 10.1093/jxb/erp112 PubMed PMID: WOS:000266348800006. 19395388

[59] Pallozzi E, Tsonev T, Marino G, Copolovici L, Niinemets Ü, Loreto F, et al Isoprenoid emissions, photosynthesis and mesophyll diffusion conductance in response to blue light. Environ Exp Bot. 2013;95(0):50–8. 10.1016/j.envexpbot.2013.06.001

[60] Kaldenhoff R. Mechanisms underlying CO_2_ diffusion in leaves. Curr Opin Plant Biol. 2012;15(3):276–81. 10.1016/j.pbi.2012.01.011 PubMed PMID: WOS:000305710300008. 22300606

[61] Terashima I, Hanba YT, Tazoe Y, Vyas P, Yano S. Irradiance and phenotype: comparative eco-development of sun and shade leaves in relation to photosynthetic CO_2_ diffusion. J Exp Bot. 2006;57(2):343–54. 10.1093/jxb/erj014 PubMed PMID: WOS:000234436700010. 16356943

[62] Flexas J, Ribas-Carbo M, Hanson DT, Bota J, Otto B, Cifre J, et al Tobacco aquaporin NtAQP1 is involved in mesophyll conductance to CO_2_ in vivo. Plant J. 2006;48(3):427–39. PubMed PMID: WOS:000241240300009. 17010114 10.1111/j.1365-313X.2006.02879.x

[63] Uehlein N, Sperling H, Heckwolf M, Kaldenhoff R. The *Arabidopsis* aquaporin PIP1:2 rules cellular CO_2_ uptake. Plant Cell and Environment. 2012;35(6):1077–83. 10.1111/j.1365-3040.2011.02473.x PubMed PMID: WOS:000303052500006. 22150826

[64] Heckwolf M, Pater D, Hanson DT, Kaldenhoff R. The *Arabidopsis thaliana* aquaporin AtPIP1;2 is a physiologically relevant CO2 transport facilitator. Plant J. 2011;67(5):795–804. 10.1111/j.1365-313X.2011.04634.x PubMed PMID: WOS:000294827700005. 21564354

[65] Terashima I, Ono K. Effects of HgCl2 on CO_2_ dependence of leaf photosynthesis: evidence indicating involvement of aquaporins in CO_2_ diffusion across the plasma membrane. Plant and Cell Physiology. 2002;43(1):70–8. 11828024 10.1093/pcp/pcf001

[66] Evans JR, Kaldenhoff R, Genty B, Terashima I. Resistances along the CO_2_ diffusion pathway inside leaves. J Exp Bot. 2009;60(8):2235–48. 10.1093/jxb/erp117 19395390

[67] Maurel C, Verdoucq L, Luu D-T, Santoni V. Plant aquaporins: membrane channels with multiple integrated functions. Annu Rev Plant Biol. 2008;59:595–624. 10.1146/annurev.arplant.59.032607.092734 18444909

[68] Aroca R, Ferrante A, Vernieri P, Chrispeels MJ. Drought, abscisic acid and transpiration rate effects on the regulation of PIP aquaporin gene expression and abundance in *Phaseolus vulgaris* plants. Ann Bot. 2006;98(6):1301–10. 10.1093/aob/mcl219 17028296 PMC2803586

[69] Xu C, Wang M, Zhou L, Quan T, Xia G. Heterologous expression of the wheat aquaporin gene TaTIP2;2 compromises the abiotic stress tolerance of *Arabidopsis thaliana*. Plos One. 2013;8(11):e79618 10.1371/journal.pone.0079618 PubMed PMID: WOS:000326503400131. 24223981 PMC3817133

[70] Grondin A, Rodrigues O, Verdoucq L, Merlot S, Leonhardt N, Maurel C. Aquaporins contribute to ABA-triggered stomatal closure through OST1-mediated phosphorylation. Plant Cell. 2015;27(7):1945–54. 10.1105/tpc.15.00421 PubMed PMID: WOS:000359358600013. 26163575 PMC4531361

[71] Shatil-Cohen A, Attia Z, Moshelion M. Bundle-sheath cell regulation of xylem-mesophyll water transport via aquaporins under drought stress: a target of xylem-borne ABA? The Plant Journal. 2011;67(1):72–80. 10.1111/j.1365-313X.2011.04576.x 21401747

[72] Zhou S, Hu W, Deng X, Ma Z, Chen L, Huang C, et al Overexpression of the wheat aquaporin gene, TaAQP7, enhances drought tolerance in transgenic tobacco. Plos One. 2012;7(12):e52439 10.1371/journal.pone.0052439 PubMed PMID: WOS:000312794500170. 23285044 PMC3527513

[73] Tazoe Y, Santrucek J. Superimposed behaviour of *g*_m_ under ABA-induced stomata closing and low CO_2_. Plant, Cell and Environment. 2015;38:385–7. 10.1111/pce.12437 25158891

[74] Tazoe Y, Von Caemmerer S, Estavillo GM, Evans JR. Using tunable diode laser spectroscopy to measure carbon isotope discrimination and mesophyll conductance to CO_2_ diffusion dynamically at different CO_2_ concentrations. Plant, Cell and Environment. 2011;34(4):580–91. 10.1111/j.1365-3040.2010.02264.x 21251018

[75] Flexas J, Bota J, Escalona JM, Sampol B, Medrano H. Effects of drought on photosynthesis in grapevines under field conditions: an evaluation of stomatal and mesophyll limitations. Funct Plant Biol. 2002;29(4):461–71. 10.1071/PP01119 32689491

[76] Trejo C, Davies WJ. Drought-induced closure of *Phaseolus vulgaris* L. stomata precedes leaf water deficit and any increase in xylem ABA concentration. J Exp Bot. 1991;42(12):1507–16.

[77] Greenway H, Gibbs J. Mechanisms of anoxia tolerance in plants. II. Energy requirements for maintenance and energy distribution to essential processes. Funct Plant Biol. 2003;30(10):999–1036. 10.1071/PP98096 32689085

[78] Gehring CA, Irving HR, Parish RW. Effects of auxin and abscisic acid on cytosolic calcium and pH in plant cells. Proceedings of the National Academy of Sciences. 1990;87(24):9645–9. 10.1073/pnas.87.24.9645 PMC55229 11607135

[79] Wilkinson S, Davies WJ. Xylem sap pH increase: A drought signal received at the apoplastic face of the guard cell that involves the suppression of saturable abscisic acid uptake by the epidermal symplast. Plant Physiol. 1997;113(2):559–73. PubMed PMID: ISI:A1997WH57200030. 12223626 10.1104/pp.113.2.559 PMC158172

[80] Tournaire-Roux C, Sutka M, Javot H, Gout E, Gerbeau P, Luu D-T, et al Cytosolic pH regulates root water transport during anoxic stress through gating of aquaporins. Nature. 2003;425(6956):393–7. http://www.nature.com/nature/journal/v425/n6956/suppinfo/nature01853_S1.html. 14508488 10.1038/nature01853

[81] Uehlein N, Otto B, Hanson DT, Fischer M, McDowell N, Kaldenhoff R. Function of *Nicotiana tabacum* aquaporins as chloroplast gas pores challenges the concept of membrane CO_2_ permeability. The Plant Cell. 2008;20(3):648–57. 10.1105/tpc.107.054023 18349152 PMC2329941

